# Deciphering the connection: Diabetes, pericyte dysfunction, and their impact on cardiovascular health

**DOI:** 10.1111/1753-0407.13539

**Published:** 2024-02-25

**Authors:** Robert Chilton, Elham I. Iranpour, Zachary Bloomgarden

**Affiliations:** ^1^ Medicine University Texas Health Science Center Houston Texas USA; ^2^ Cardiology Division University of Texas Health Science Center at San Antonio San Antonio Texas USA; ^3^ Department of Medicine, Division of Endocrinology, Diabetes and Bone Disease Icahn School of Medicine at Mount Sinai New York New York USA

In the intricate tapestry of human biology, diabetes mellitus silently threads its way, casting unseen webs across the expanse of global health.^1^ This enduring metabolic disorder, characterized by elevated blood glucose levels, poses a challenge that extends beyond its well‐documented impact on blood vessels. Unbeknownst to many, diabetes conducts a symphony of disruption, particularly affecting the custodians of vascular harmony – the pericytes.

Known alternately as Rouget cells or perivascular cells, these pericytes are not mere passive spectators in the Grand Theater of the human body. Enveloping the microvascular basement membrane throughout the body, they were first perceived as structural support for blood vessels.^2^ However, as the pages of scientific exploration turn, their dynamic nature emerges, revealing involvement in crucial processes such as angiogenesis, vascular stability, and immune response modulation.^3^

The cardiovascular system, a delicate ballet of physiologic processes, relies on the intricate equilibrium between endothelial cells and pericytes for its integrity. Pericytes, in their pivotal role, regulate blood flow, vessel permeability, and tissue perfusion, maintaining vascular function.^4^, ^5^

This delicate balance, however, is disrupted by hyperglycemia, the hallmark of diabetes, which casts a malevolent shadow, triggering events that wreak havoc on pericyte function. Oxidative stress emerges as a primary antagonist, inducing the overproduction of reactive oxygen species that compromises pericyte support in maintaining vascular stability. Inflammation, driven by the relentless onslaught of hyperglycemia, causes phenotypic changes in pericytes, compromising their ability to regulate vascular tone and permeability.^6^ Advanced glycation end‐products (AGEs) further disrupt the once harmonious communication between pericytes and endothelial cells, throwing the entire cardiovascular system into disarray.^7^, ^8^

The symphony of pericyte–endothelial crosstalk, once melodious, now falters in the face of diabetes. Dysregulated angiogenesis leads to abnormal vessel formation and impaired tissue perfusion, casting a dark shadow over the previously vibrant landscape of vascular homeostasis. Capillary rarefaction ensues, reducing capillary density and exacerbating organ damage, especially in highly vascularized organs like the heart.^9^

Yet, amidst the chaos, potential opportunities for new treatment approaches emerge. Pericytes, it seems, play a crucial role in the saga of myocardial ischemia. Studies suggest their involvement in inflammation modulation, angiogenesis, and the regeneration of the infarcted heart.^10^ The flicker of potential therapeutic avenues suggests that pericytes might hold the key to unraveling the mysteries of cardiovascular regenerative medicine.^11^

In addition, these specialized cells are also found in the microvasculature of both retinal and renal systems, again playing roles in the vascular complications of diabetes.^12^ In the retina, pericytes are essential for the formation and maintenance of the blood–retinal barrier (BRB).^13^ The BRB regulates the passage of nutrients and ions between the blood and the neural tissue of the retina, ensuring a stable microenvironment crucial for optimal visual function. Pericytes contribute to the structural support of capillaries, regulate blood flow, and participate in the maintenance of vascular permeability, thus preventing leakage that could lead to vision‐threatening conditions such as diabetic retinopathy.^14^

Correspondingly, in the kidneys, pericytes are key players in the regulation of renal blood flow and glomerular filtration.^15^ They are integral components of the renal vasculature, contributing to the structural integrity of capillaries in the glomerulus. Pericytes modulate blood pressure in the kidneys, affecting the filtration of waste products and maintenance of electrolyte balance. Dysfunction of pericytes in the renal system is associated with various renal disorders, including diabetic nephropathy.

In summation, pericytes are indispensable in preserving the normal functioning of the retina and kidneys, highlighting their pivotal role in maintaining vascular homeostasis and preventing the onset of debilitating ocular and renal conditions.

As the scientific tale unfolds, it becomes evident that understanding the impact of diabetes on pericyte function opens doors to potential therapeutic interventions. Antioxidant therapies, anti‐inflammatory compounds, and strategies targeting AGE formation become the knights in shining armor, venturing to preserve pericyte function and reduce the looming threat of diabetes‐related cardiovascular complications.^2^

**FIGURE 1:**
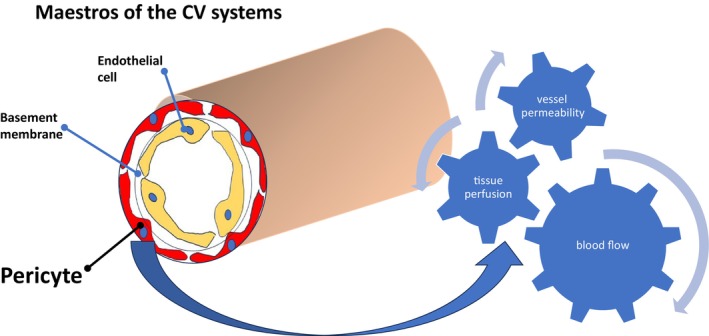
The intricate network of endothelial cells, composing a singular cell layer, envelops the entirety of blood vessels, overseeing the intricate ballet of exchanges between the bloodstream and the neighboring tissues. These endothelial cells serve as conductors, orchestrating signals that harmonize the growth and maturation of connective tissue cells, shaping the layers that cocoon the blood‐vessel wall. In essence, the endothelial cells emerge as master architects, intricately choreographing the symphony of cellular interactions essential for the structure and function of the vascular system. Pericytes envelop the outer surface of the endothelial tube using cytoplasmic extensions. They collaborate with endothelial cells in generating a common basement membrane, underscoring the crucial role of their interaction in shaping, sustaining, and altering the basement membrane. Positioned closely to endothelial cells, pericytes are usually around 20 nanometers away and partially cover multiple endothelial cells.^16^ CV, cardiovascular.

The development of novel therapeutic modalities offers the potential to enhance angiogenesis and microvascular perfusion. The intricate connection between diabetes and cardiovascular disease, illuminated by the critical role of pericytes, hints at a future where innovative approaches could revolutionize the management of cardiovascular complications in people battling the relentless adversary that is diabetes (Figure 1).
